# Supplementary material for: Design of Trials for Cerebral Small Vessel Disease and Vascular Cognitive Impairment
Source: Neurol Int. 2025 Nov 4;17(11):181. doi: 10.3390/neurolint17110181 (PMC12655689; doi:10.3390/neurolint17110181)
Supplement: Supplementary file 1 [file neurolint-17-00181-s001.zip › Supplementary_FileS1_Search strategy.pdf]

1. **cSVD Search**

1.1 Ovid MEDLINE and EMBASE searches

**Ovid MEDLINE and EMBASE**	
**Step**	**Terms**
1	Randomized Controlled Trials as Topic/
2	Multicenter study.pt.
3	Exp Clinical Trials as topic/
4	((singl$ or doubl$ or treb$ or tripl$) adj (blind$3 or mask$3)).tw.
5	PLACEBOS/
6	Randomly allocated.tw.
7	(allocated adj2 random$).tw.
8	1 or 2 or 3 or 4 or 5 or 6 or 7
9	Cerebral small vessel disease
10	cSVD
11	Binswanger*
12	(cerebral adj arteriosclerosis)
13	(cerebral adj microbleeds)
14	(cerebral adj microinfarcts)
15	Lacunar stroke
16	Lacunar infarct
17	(small vessel adj2 dis*)
18	9 or 10 or 11 or 12 or 13 or 14 or 15 or 16 or 17
19	8 and 18
20	Limit 19 to human
21	Limit 20 to humans
22	Limit 21 to yr="2012-Current"

1.2 ClinicalTrials.gov Advanced Search

**Search filter**	**Content**
Condition or disease	Cerebral small vessel disease
Other terms	cSVD OR Binswanger OR Lacunar stroke OR Cerebral small vessel ischaemic disease OR Lacunar infarct OR Subcortical vascular dementia OR Cerebral microbleed OR Cerebral microinfarct OR Lacune
Study type	Interventional Studies (Clinical Trials)
Study Results	All Studies
Status	Recruitment: Not yet recruiting, Recruiting, Enrolling by invitiation, Active, Suspended, Terminated, Withdrawn, Unknown status

All other search filters were left empty.

1.3 European Union Clinical Trials Register Search

Search terms: Cerebral small vessel disease OR cSVD OR Binswanger OR Lacunar stroke OR Cerebral small vessel ischaemic disease OR Lacunar infarct OR Subcortical vascular dementia OR Subcortical

ischaemic vascular dementia OR Cerebral microbleed OR Cerebral microinfarct OR Lacune OR Small vessel disease

All other search filters were left empty.

1.4 International Clinical Trials Registry Platform Advanced Search

Search filter	Content
Title	Cerebral small vessel disease OR cSVD OR Binswanger OR Lacunar stroke OR Cerebral small vessel ischaemic disease OR Lacunar infarct OR Subcortical vascular dementia OR Subcortical ischaemic vascular dementia OR Cerebral microbleed OR Cerebral microinfarct OR Lacune OR Small vessel disease
(OR) Condition	Cerebral small vessel disease OR cSVD OR Lacunar stroke OR Subcortical stroke OR Lacunar infarction OR Subcortical infarction OR Subcortical vascular dementia OR Subcortical ischaemic vascular dementia OR Small vessel disease
Recruitment status	ALL

All other search filters were left empty.

2. <u>VCI Search</u>

2.1 Ovid MEDLINE and EMBASE searches

Ovid MEDLINE and EMBASE	
**Step**	**Terms**

1	exp Dementia, Multi-Infarct/
2	exp Dementia, Vascular/
3	“subcortical ischemic vascular disease*”.ti,ab.
4	“vascular cognitive impairment*”.ti,ab.
5	“vascular dement*”.ti,ab.
6	VaD.ti,ab.
7	VCI.ti,ab.
8	1 or 2 or 3 or 4 or 5 or 6 or 7
9	Randomized Controlled Trials as Topic/
10	Multicenter study.pt.
11	exp Clinical Trials as topic/
12	((singl$ or doubl$ or treb$ or tripl$) adj (blind$3 or mask$3)).tw.
13	PLACEBOS/
14	Randomly allocated.tw.
15	(allocated adj2 random$).tw.
16	9 or 10 or 11 or 12 or 13 or 14 or 15
17	8 and 16
18	remove duplicates from 17
19	limit 18 to human
20	limit 19 to humans
21	Limit 20 to yr=”2012 – 2022”

2.2 PsycInfo (EBSCO) Basic Search

**Vascular dementia search**	
Search Terms	‘Vascular dementia’ AND trial
Limiters	2012-2022, Peer-reviewed journals, Humans
**VCI search**	
Search Terms	‘Vascular cognitive impairment’ AND trial
Limiters	2012-2022, Peer-reviewed journals, Humans

2.3 ClinicalTrials.gov Advanced Search

**Search filter**	**Content**
Condition or disease	‘Vascular dementia’ for VaD studies<br>‘Vascular cognitive impairment’ for VCI studies
Other terms	Left blank
Study type	Interventional Studies (Clinical Trials)
Study Results	All Studies
Status	Recruitment: Not yet recruiting, Recruiting, Enrolling by invitiation, Active, Suspended, Terminated, Withdrawn, Unknown status

All other search filters were left empty.

2.4 European Union Clinical Trials Register Search

Search terms: ‘Vascular dementia’ (for VaD studies), ‘Vascular cognitive impairment’ (for VCI studies)

All other search filters were left empty.

2.5 International Clinical Trials Registry Platform Advanced Search

**Search filter**	**Content**
**Vascular dementia search**	
Title	Vascular dementia
(OR) Condition	Vascular dementia
Recruitment status	ALL
**VCI search**	
Title	Vascular cognitive impairment
(OR) Condition	Vascular cognitive impairment
Recruitment status	ALL

All other search filters were left empty.
