# Supplementary material for: Design of Trials for Cerebral Small Vessel Disease and Vascular Cognitive Impairment
Source: Neurol Int. 2025 Nov 4;17(11):181. doi: 10.3390/neurolint17110181 (PMC12655689; doi:10.3390/neurolint17110181)
Supplement: Supplementary file 1 [file neurolint-17-00181-s001.zip › Supplementary_FileS2_Data Dictionary.pdf]

Data Dictionary for Categories used in Data Extraction

No.	Variable	Definition
**INCLUSION AND EXCLUSION CRITERIA**		
1.1	Cognition	Selection criteria relating to cognitive parameters and/or symptoms. These might include a cut-off score for cognitive test, presence of cognitive impairment or isolated symptoms. Examples of cognitive tests include the mini-mental state examination (MMSE), clinical dementia rating (CDR) , Montreal Cognitive Assessment (MoCA), Hasegawa's Dementia Scale (HDS-R), etc.<br><br>For instance, a patient may be included into a trial for having MoCA score <27 or excluded for having a MoCA score <18.
1.2	Comorbidities	Non-vascular comorbidities usually listed as exclusion criteria, which may affect the trial protocol or results. These may include:<br>• Neurological diseases apart from vascular cognitive impairment or vascular dementia, including non-vascular dementias (e.g. Multiple sclerosis, Alzheimer's disease, Frontotemporal dementia etc.)<br>• Gastrointestinal disease<br>• Endocrinological or metabolic disease<br>• Cancer or terminal/life-threatening illness<br>• Any other non-vascular comorbidities
1.3	Functional status	Patients' functional status, measured clinically or using scoring scales. This includes scales measuring levels of impairment, activity, participation, and quality of life. (e.g. Modified Rankin Scale, National Institute of Health Stroke Scale, Barthel Index, Activities of Daily Living)<br><br>Cognitive and motor impairments are not included under 'Functional status'. These are listed under the following categories:<br>*Cognitive impairment = 'Cognition'<br>*Motor impairment = 'Neurological impairments'
1.4	Hachinski Ischaemic Scale	*Only used in VaD/VCI Trials section<br>The Hachinski Ischaemic Scale (HIS) is a clinical tool used to differentiate Alzheimer's disease and vascular dementia by measuring the number and severity of a patient's vascular risk factors and clinical features of cerebrovascular disease. A high HIS score in a dementia patient indicates a high probability of vascular dementia.

1.5	Neuroimaging	Neuroimaging features specific to chronic cerebrovascular disease may form part of inclusion criteria (e.g. White matter hypertensities, Cerebral microbleeds, Lacunes, Subcortical infarcts, Perivascular space, Brain atrophy)<br><br>Some trials may use neuroimaging features of other pathologies as exclusion criteria (e.g. Large artery or cortical strokes, Subarachnoid haemorrhage)
1.6	Neurological impairments	Neurological impairments caused by cerebrovascular disease, including ongoing stroke impairments (e.g. Motor weakness, sensory losses, aphasia, incontinence, sleep disturbances, etc.). Neurological impairments may also refer to stroke impairments measured by the National Institute of Health Stroke Scale (NIHSS).
1.7	Neuropsychiatric & Mood Disorders	Selection criteria relating to neuropsychiatric and mood-related symptoms, disorders or score-based assessments. Neuropsychiatric features commonly seen in cerebral small vessel disease and dementia include depression, anxiety, irritability, apathy, delusions, hallucinations, disinhibition etc. Neuropsychiatric and mood assessments may include Neuropsychiatric Inventory-Clinician rating scale (NPI-C), Hamilton Depression Scale (HAM-D), Cornell Scale for Depression in Dementia (CSDD), Beck Depression Inventory, etc.
1.8	Physiological Measurements	Cut-off numbers in physiological measurements used as selection criteria. Examples may include:<br>• Haemodynamic and vascular measurements (e.g. Heart rate, Blood pressure, Peripheral arterial tone, Arterial stiffness)<br>• Electroencephalography (EEG) or other electrophysiological measurements (e.g. Motor evoked potentials, Corticomotor excitability)
1.9	Stroke Occurrence	Clinical evidence or past history of stroke.
1.10	Tissue / Plasma / CSF Biomarkers	Tissue, plasma or CSF biomarkers related to cerebrovascular disease (e.g. Blood lipids, C-reactive protein, Glucose, etc.)
1.11	Vascular risk factors	Vascular comorbidities or risk factors that may be listed as part of the selection criteria, which may explain cognitive impairment or affect the trial protocol or results (e.g. Genetic causes of cerebrovascular disease, Carotid artery stenosis, Peripheral artery disease, Cardiovascular disease, Hypertension, Diabetes mellitus etc.)

INTERVENTIONS		
2.1	Antihypertensives	Drugs that reduce systemic blood pressure
2.2	Antithrombotics	Drugs that reduce and prevent the formation of thrombi or blood clots. These may include:<br>• Antiplatelets<br>• Anticoagulants<br>• Thrombolytic drugs
2.3	Cognitive Enhancers / Anti-Dementia Drugs	Drugs that improve cognitive function (memory, arousal, attention, concentration, etc.) These may include:<br>• Anticholinergic drugs<br>• NMDA receptor antagonists<br>• Dopamine agonists / Dopamine reuptake inhibitors
2.4	Cognitive Rehabilitation	Non-pharmacological therapies aimed at improving cognitive function. These may include:<br>• Attention processing training<br>• Speech language therapy<br>• Occupational therapy<br>• Problem-solving games<br>• Motor dexterity tasks
2.5	Exercise & Lifestyle Interventions	Lifestyle and exercise interventions aimed at improving neurological function and/or reducing risk factors to treat cerebrovascular disease. These may include:<br>• Exercise programmes (e.g. High-intensity aerobic exercise, Yoga, Weight training)<br>• Dietary modifications<br>• Stress management / Relaxation techniques
2.6	Physiological Interventions	Non-pharmacological interventions which involve physical training or altering physiology. These may include:<br>• Physiotherapy / Physical rehabilitation (e.g. Gait and balance training)<br>• Ischaemic conditioning<br>• Hyperbaric oxygen therapy<br>• Electrophysiological or magnetic stimulation of the brain
2.7	Traditional Chinese Medicine	Traditional Chinese Medicine (TCM) is an alternative medicine practice originating from China involving a wide range of medicinal products, devices and therapies. TCM has been proposed as a potential treatment for cognitive impairment in cerebral small vessel disease. TCM

		interventions usually have multiple pharmacological targets that may lead to the attenuation of symptoms in cerebral small vessel disease, such as increased cerebral blood flow, reduced production of pro-inflammatory and increased neurogenesis.<br><br>TCM in this study largely refers to medicinal products and devices. Physical TCM therapies, such as tai chi, are classified under 'Exercise & Lifestyle Interventions'.
2.8	Vasoactive Drugs	Drugs that alter vascular/endothelial activity, leading to changes in haemodynamic function, such as blood pressure, heart rate, systemic vascular resistance, stroke volume, heart contractility etc. These may include:<br>• Phosphodiesterase inhibitors<br>• Nitrates<br>• Adrenergic receptor agonists / antagonists<br>Inotropic agents
**OUTCOMES**		
3.1	Clinician assessment	Tests or assessments involving clinician impression (e.g. Clinical Global Impression of Change, Clinician's Interview-Based Impression of Change Plus caregiver input)
3.2	Cognition	Changes in cognitive deficits or cognitive test scores (e.g. MMSE, CDR, MoCA)
3.3	Economic outcomes	Outcomes related to economic evaluation, including resource use (e.g. hospital admissions, visits to specialists/GP, emergency visits, medications) and cost-effectiveness
3.4	Functional outcomes	Patients' functional scores or outcomes. This included scales that measure impairment, activity, participation and quality of life. (e.g. Modified Rankin scale, Barthel Index, Activities of Daily Living)
3.5	Neuroimaging (Other)	Non-structural brain imaging outcomes. These may include haemodynamic, chemical or functional changes caused by cerebrovascular disease leading to vascular cognitive impairment or dementia, measured using radiological modalities (e.g. Cerebrovascular reactivity, Transcranial Doppler ultrasound, Functional magnetic resonance imaging)
3.6	Neuroimaging (Structural)	Structural brain imaging outcomes. These may include changes or damage to the gross anatomy of the brain caused by cerebrovascular disease leading to vascular cognitive impairment or dementia, visualized using radiological modalities (e.g. Magnetic resonance imaging, Computed tomography)

3.7	Neurological impairments	Changes in neurological function and/or signs and symptoms, as for example, measured by the National Institute of Health Stroke Scale (NIHSS)
3.8	Neuropsychiatric & Mood Disorders	Changes in mood or neuropsychiatric scores e.g. Hamilton Depression Scale (HAM-D), Cornell Scale for Depression in Dementia (CSDD), Beck Depression Inventory (BDI), Neuropsychiatric Inventory-Clinical Rating (NPI-C), etc.
3.9	Physiological Measurements	Changes in physiological measurements. Examples may include:<br>• Haemodynamic and vascular measurements (e.g. Heart rate, Blood pressure, Peripheral arterial tone, Arterial stiffness)<br>• Electroencephalography (EEG) or other electrophysiological measurements (e.g. Motor evoked potentials, Corticomotor excitability)
3.10	Safety & Adherence Outcomes	Safety outcomes, including adverse events, death, and treatment adherence and tolerability
3.11	Stroke Occurrence	Occurrence or recurrence of a clinical stroke event
3.12	Tissue / Plasma / CSF Biomarkers	Tissue, plasma or CSF biomarkers related to cerebrovascular disease (e.g. Blood lipids, C-reactive protein, Glucose, etc.)
